# Written-Word Concreteness Effects in Non-attend Conditions: Evidence From Mismatch Responses and Cortical Oscillations

**DOI:** 10.3389/fpsyg.2018.02455

**Published:** 2018-12-13

**Authors:** Dawei Wei, Margaret Gillon-Dowens

**Affiliations:** ^1^School of Foreign Languages, Lanzhou Jiaotong University, Lanzhou, China; ^2^Cognitive Neuroscience of Language Laboratory, University of Nottingham Ningbo China, Ningbo, China

**Keywords:** concreteness effect, visual mismatch negativity (vMMN), Chinese single-character words, timefrequency (TF) analysis, theta power increase, alpha power decrease, phase locking

## Abstract

It has been widely reported that concrete words have processing advantages over abstract words in terms of speed and efficiency of processing, a phenomenon known as the concreteness effect. However, little is still known about the early time-course of processing concrete and abstract words and whether this concreteness effect can still persist in conditions where attention is not focused on the words presented (automatic processing). This study aimed to shed light on these issues by examining the electrophysiological brain responses to concrete and abstract words. While participants were engaged in a non-linguistic color tracking task presented in the center of the monitor screen, matched Chinese concrete and abstract single-character words appeared within a passive oddball paradigm, out of the focus of attention. In calculating visual Mismatch Negativity (vMMN), Event-related potentials (ERPs) to words of the same semantic category were compared when these words were presented as deviants and standards. Before 320 ms, both abstract and concrete words yielded vMMN with left-lateralized distribution, suggesting similar verbal processing at an initial processing stage. After 320 ms, only concrete words additionally elicited vMMN with a central distribution. Time frequency (TF) analysis of the results also revealed larger theta power increase (200–300 ms) and theta power phase locking (200–450 ms) for concrete than for abstract words. Interestingly, there was more alpha power decrease for abstract than for concrete words from 300 to 450 ms. This may reflect the greater difficulty in processing abstract meaning. Taken together, our ERP and TF results point to the existence of different neural mechanisms underlying non-attentive processing of abstract and concrete words.

## Introduction

The linguistic concreteness effect refers to the processing advantage of words representing imaginable, concrete concepts (e.g., apple) over those representing abstract concepts without sensory referents (e.g., skill). Specifically, in various experimental tasks such as lexical decision tasks, concrete words are often found to be recognized more quickly and more completely and remembered more accurately than abstract words (e.g., Strain et al., 1995; Peters and Daum, 2008; Barber et al., 2013). The concreteness effect has been of central interest among linguists and psychologists, as its sharp contrast between two semantic categories has been explored as a window onto the nature and structure of semantic representation in the human mind (Dalla Volta et al., 2014). Among the many semantic theories put forward to explain this effect, there are two predominant and competing ones, namely, the dual-coding theory (Paivio and Begg, 1971; Paivio, 1991) and the context availability theory (Schwanenflugel and Shoben, 1983; Schwanenflugel and Stowe, 1989). The dual coding theory proposes two qualitatively distinct systems of semantic processing, one verbal and the other non-verbal and imagery-based. Although both concrete and abstract words share the verbal processing system, concrete words can additionally engage the perceptual, imagery-focused system, which results in processing advantages. In comparison, the context availability theory argues for a common verbal system for both types of words but that concrete words can activate more associative information, resulting in a processing advantage over abstract words.

Based on clinical reports (Coltheart et al., 1980; Coslett and Saffran, 1989) and behavioral visual hemifield stimulation (Day, 1979; Chiarello et al., 1987), it has been argued that the verbal system is left-lateralized, while the imagery-based system is represented in the right hemisphere. So from a neural perspective, the dual coding theory predicts more involvement of right hemisphere for concrete than for abstract words. The context availability theory, in contrast, predicts that concrete words will have stronger activation than abstract words only in the left hemisphere. In recent decades, an increasing number of neuroimaging studies using functional Magnetic Resonance Imaging (fMRI) and Positron Emission Topography (PET) have investigated the neural substrates of concrete and abstract words. The findings from these studies are quite diverse. Some investigations have found more involvement of left hemisphere for concrete words (Jessen et al., 2000; Fiebach and Friederici, 2004), other studies have reported increased activity in the left hemisphere for abstract words, and bilateral activity for concrete words (Binder et al., 2005; Sabsevitz et al., 2005). Still others have described more activity only for abstract words (Kiehl et al., 1999; Perani et al., 1999; Noppeney and Price, 2004). In light of the inconsistent findings, it seems there is no clear consensus at present about the neural mechanisms of the concreteness effect.

In addition to metabolic neuroimaging studies, the event-related potential (ERP) technique has been widely used to explore this issue. One advantage of this technique is its millisecond-level time resolution, which is critical in uncovering details of when the cognitive processing of different stimulus events diverges. Findings from ERP studies can also be used to distinguish between various cognitive processes and representations based on the “spatial distinctiveness principle” (Holcomb et al., 1999). This principle assumes that heterogeneous cognitive processes tend to be associated with separate spatial distributions to a greater extent than will a single homogeneous process. Therefore, different topographical ERP patterns should be expected if concrete and abstract words are processed differently. Across different languages and a variety of tasks, such as explicit abstractness/imageability judgments and lexical decision, concrete words elicit larger N400 than abstract words, with a topographical distribution in posterior and anterior areas (Holcomb and Anderson, 1993; Holcomb et al., 1999; West and Holcomb, 2000; Tolentino and Tokowicz, 2009; Tsai et al., 2009). For example, using a lexical decision task, Zhang et al. (2006) investigated the neural dynamics of concrete and abstract Chinese words. Their findings replicated the typical concreteness N400 effect reported in other ERP studies (e.g., West and Holcomb, 2000). Concrete and abstract word effects also presented different scalp distributions, which was taken as supporting evidence for the dual-coding theory.

While most previous ERP studies have focused on the time interval of the N400 component, that is, 350–500 ms after stimulus onset, the dynamics of semantic processing at early latencies before 300 ms remain largely unexplored. Some recent studies, however, have provided evidence that semantic processing can be detected within the first 250 ms post-stimulus (Moscoso Del Prado Martín et al., 2006; Glenberg et al., 2008; Pulvermüller et al., 2009). Among the few ERP studies exploring early differences between abstract and concrete words, Wirth et al. (2008) investigated how a single-word context influenced the processing of abstract and concrete words. They manipulated concreteness (abstract vs. concrete words) and semantic relatedness (related vs. unrelated). In an early P1/N1 latency range, related as compared to unrelated abstract words were activated more in the left prefrontal cortex. For concrete words, this context effect was diminished, and abstract and concrete words performed differently according to the semantic context at a very early stage. Some other studies compared abstract and concrete words in terms of their elicitation of the recognition potential (RP) (Rudell, 1992) in a lexical decision task. RP is an early (before 300 ms) negative deflection sensitive to lexical-semantic manipulation (Hinojosa et al., 2004). In their study (Martiìn-Loeches et al., 2001), concrete words elicited larger RP than abstract words, but there were no differences in terms of the topographical distribution of RP. The authors interpreted this result as supporting a single unitary semantic system for both word types. Given that this result does not agree with previous N400 studies showing differences between abstract and concrete words, the authors suggested that, in their study, the similar processing mechanism for both word types may be confined to the early latency. Clearly, in order to have a complete picture of the differences between abstract and concrete word processing, it is necessary to find an approach which can focus on semantic processing at both early and late time windows. Another noteworthy point is that previous studies typically have used language-related tasks such as lexical decision and semantic categorization. With these tasks, participants need to pay active, conscious attention to the lexico-semantic stimuli in order to engage semantic analysis as expected. This, however, raises the question as to whether the N400 concreteness effect will still persist in non-attend conditions. A positive answer to this question can thus provide additional evidence for the concreteness effect, since the advantage would be generated by concrete words *per se* and independently of top-down task effects. In fact, a few studies have found that semantic processing cannot only start early but can occur automatically, that is, without focused attention (Pulvermüller et al., 2005; Relander et al., 2009).

To answer these questions and fill the present research gaps in the area, the current study will focus on (visual) Mismatch Negativity (v)MMN as a critical ERP component in investigating the concreteness effect. MMN has long been demonstrated to be a reliable index of early and automatic processing of stimulus change (Näätänen, 2008). Its applications in spoken language studies are well documented (Pulvermüller and Shtyrov, 2006). Its early latency and sensitivity to semantic change (Shtyrov and Pulvermüller, 2007) thus make it an attractive tool for the current investigation into processing differences between abstract and concrete words. Numerous studies have found that the MMN component is sensitive to changes in speech features such as lexicality and semantics, as well as basic auditory attributes (e.g., acoustic duration, for a review see Pulvermüller and Shtyrov, 2006). For example, real words have been shown to elicit enhanced MMN compared to psycholinguistically matched pseudowords (e.g., Korpilahti et al., 2001; Kujala et al., 2007; Gu et al., 2012). Early semantic access has also been documented using an MMN approach. Shtyrov et al. (2004) compared MMN responses to two English verbs involving different body parts, the hand-related verb *pick* and leg-related verb *kick* in an oddball paradigm. Neuronal distribution of “pick” was found to be more lateral while that of “kick” more focal. This topographical difference seems to reflect different sensorimotor somatotopy rooted in the word semantics. In addition, recognition of the words started as early as 140–180 ms. The different activation patterns clearly demonstrate early, automatic semantic access to spoken words. These lexical and semantic MMN effects can be best explained by the long-term memory trace theory proposed by Pulvermüller and Shtyrov (2006). According to this theory, through associative language learning experience over time, long-term memory representations of existing words and their semantic features can be developed and shaped at the level of the neuronal network. These representations or traces over time become sufficiently robust to bolster rapid and automatic response to the word features, even in an attention-deprived condition (Shtyrov and Pulvermüller, 2007). Such responses can be neurophysiologically reflected as MMN effects (Pulvermüller and Shtyrov, 2006).

In recent years, MMN in the visual domain has also been revealed as an indicator of early visual change detection of elementary visual features such as color (Czigler et al., 2002), abstract sequential regularities (Stefanics et al., 2011) and even higher-level linguistic changes (Files et al., 2013; Shtyrov et al., 2013; Wang et al., 2013). In a passive oddball paradigm where matched Chinese single-character real words and pseudowords were compared, Wei et al. (2018) found that in native readers, only real words elicited vMMN as early as around 200 ms. In contrast, there was no sign of vMMN to either real words or pseudowords in a non-native control group with little learning experience in the Chinese language. This study largely replicated the lexical MMN findings in the auditory modality, thus supporting MMN as a modality-independent early index at least for lexical processing. Therefore, these studies suggest the potential role of vMMN in studying early linguistic effects. In addition, oddball paradigms, especially the identity oddball paradigm, where MMN is typically elicited, offer another advantage in controlling the physical variance of stimuli. In an identity oddball scheme, the role of deviant and standard stimuli in one sequence is swapped in the other one. The potential MMN is measured by comparing the same stimuli acting as deviants and standards across the two sequences. Therefore, it is possible to eliminate potential physical confounding and isolate a relatively pure cognitive process of interest (Pulvermüller and Shtyrov, 2006). In fact, physical variance between concrete and abstract words may have partially led to the ambiguous findings so far reported, so the identity paradigm could have potential for overcoming this confounding factor.

Additionally, to further explore the neural mechanisms of the concreteness effect, a time-frequency (TF) analysis of the acquired electroencephalography (EEG) data will also be carried out. While the “standard” ERP data analysis is to compute averaged evoked potentials time-locked to an event of interest and compare the amplitudes and latencies as a function of time across different experimental conditions, an increasingly popular development of EEG data analysis is to uncover their oscillatory neuronal dynamics in the frequency domain. Evoked activity features an identical phase (“phase-locked”) and can be visible in averaged ERPs, whereas induced oscillations, though correlated with experimental conditions, have different onset times and/or phase jitter (“non-phase-locked”), and are therefore not visible in the time domain. However, many studies have demonstrated that both evoked and induced activities are important modes of brain functioning (Roach and Mathalon, 2008). The sum of both evoked and induced event-related oscillations is called overall spectral power or event-related spectral perturbation (ERSP). The phase-locking value/factor (PLV/PLF) or inter-trial (phase) coherence (ITC/ITPC) is another important complement to ERSP because it statistically assesses the event-related phase consistency of oscillations irrespective of their amplitude (Tallon-Baudry et al., 1997). PLV is between zero and one. If the phases during latency after the onset of a stimulus are a random distribution across trials, i.e., non-phase-locked activity, PLV would be zero. If, however, the phases are identical across all trials, i.e., strictly phase-locked activity, PLV would be one. In sum, time-frequency analyses of EEG signals provide additional information about which frequencies have the largest spectral power in a given latency and how their phases synchronize across time and space. These oscillatory dynamics have been found to be associated with various cognitive processes such as executive control, language and emotion, so time-frequency analysis can add new insights to the traditional ERP approach (Bastiaansen et al., 2012).

Neural oscillations are typically categorized into four frequency bands for analysis, delta (0–3 Hz), theta (4–7 Hz), alpha (8–12 Hz), beta (13–30 Hz, with the lower beta at 13–18 Hz), and gamma (above 30 Hz). A large corpus of studies over the last two decades has found a range of cognitive functions reflected in neuronal oscillatory dynamics in these four bands. For example, studies show that sustained gamma activity may play a critical role in holding working memory traces (Kaiser et al., 2003; Jokisch and Jensen, 2007) and encoding of long-term memory representations (Osipova et al., 2006). In contrast, suppressing interference to prioritize task-relevant operations correlates with an increase in the oscillatory activity in the posterior alpha band (Klimesch et al., 1999; Jensen et al., 2002). Additionally, the theta frequency band has also been linked with memory retrieval and working memory (Bastiaansen et al., 2005, 2008). Delta band activity is involved in decision making and signal detection, to name but two functions (Başar et al., 2001; Yordanova et al., 2004). Of particular relevance to the current investigation are studies about the oscillatory correlates of lexical-semantic processing. Previous studies have found the importance of alpha band in semantic operations (Klimesch, 1999), and gamma band in semantic memory retrieval (Pulvermüller, 1999). Recent studies also seem to underscore the critical role of theta band oscillations in lexico-semantic processing. Bastiaansen et al. (2005) compared the oscillatory dynamics of meaning-bearing open-class words (“OC,” e.g., nouns and verbs) and closed class words (“CC,” e.g., determiners, articles), which carry more syntactic information, in a story-reading experiment. Both OC and CC words were found to have theta band power increase, as well as decrease in alpha and beta ranges. However, only OC words showed more theta synchronization over left temporal areas, which is argued to function in lexico-semantic retrieval (Indefrey and Cutler, 2004).

While quite a number of studies have investigated oscillatory patterns in language comprehension and general cognitive processing, only a handful of such studies have used MMN designs. Several TF studies of auditory MMN experiments have shown the important role of theta band in the generation of mismatch oscillatory responses (MOR) (Fuentemilla et al., 2008; Hsiao et al., 2009; Ko et al., 2012). In a visual MMN study on color change detection, it was found that theta band activity played a similar role in eliciting vMMN (Stothart and Kazanina, 2013). While no difference in alpha band power between deviant and standard stimuli has been reported in auditory MMN studies (e.g., Hsiao et al., 2009; Ko et al., 2012), two studies in the visual modality have indeed found such a difference. Stothart and Kazanina (2013) and Tugin et al. (2016) both described stronger alpha power decrease induced by deviants than standards. Tugin et al. (2016) also reported a larger increase of alpha band to deviant than standard items, despite an absence of vMMN in their study. Thus, given the confirmed roles of various frequency bands in linguistic processing as well as in domain-general cognition, a TF analysis of the ERP data of the current experiment will be carried out to paint a more complete picture of any concreteness-related effects.

In sum, the current study aims to further examine the effects of concreteness by exploring elicitation of the vMMN component, using a passive oddball paradigm. Based on previous findings, it is hypothesized that both concrete and abstract words may elicit vMMN, but concrete words may yield larger vMMN than abstract words. As previously stated, very few studies have attempted to explore the neuronal oscillatory dynamics of concrete and abstract words, so no specific hypothesis can be formulated about this issue. However, based on previous TF studies on language comprehension, especially the role of theta band spectral power and alpha power decrease in semantic processing (Klimesch et al., 1999; Bastiaansen et al., 2008, 2012), it is tentatively predicted that concrete words may elicit larger theta power increase than abstract words.

## Materials and Methods

### Participants

Twenty-five neurologically and psychologically healthy college students (average age 21.5, *SD* = 1.2, male 8) participated in the experiment for course credit. They were all right-handed, native speakers of Chinese and had normal or corrected-to-normal vision and gave written informed consent in accordance with the Declaration of Helsinki before the experiment. The study was approved by the Research Ethics Committee of University of Nottingham Ningbo China and carried out in accordance with the approved regulations.

### Stimuli

The stimuli included a set of five concrete and five abstract Chinese single-character words. These words were selected from a pool of 15 concrete and 15 abstract words with similar averaged ratings in number of strokes, frequency (counts per million), phonetic regularity and concreteness, which were chosen from the online Chinese Single-character Word Database (Liu et al., 2007). Twenty college students who did not take part in the ERP experiment were invited to carry out a rating task, judging the words in terms of emotional valence, arousal, frequency and concreteness on a seven-point Likert Scale (1 meaning the least positive, arousing, frequent or concrete). Based on the rating scores and data from the database, the final 10 words, five for each semantic category, were chosen. The final two sets are significantly different in concreteness (*p* < 0.001) but similar in all the other dimensions (all *p*s > 0.1) (see Table 1 for a list of the stimuli. Numbers after the pinyin transcription represent the word tone).

**Table 1:** Two different types of character stimuli.

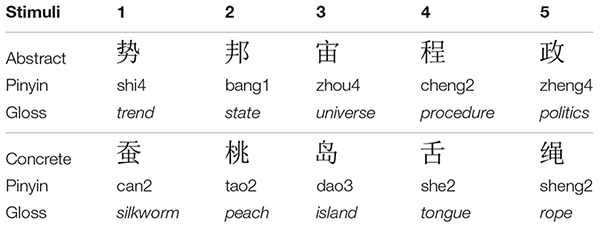

### Procedure

**FIGURE 1:**
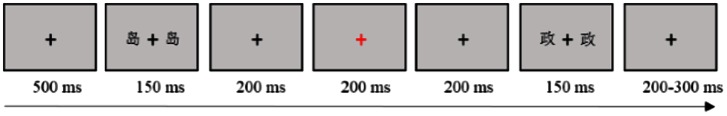
Illustration of experimental procedure.

A complete trial is shown in Figure 1. During the experiment, participants were seated in a comfortable chair at a viewing distance of 50 cm from the monitor. Their task was to press keys when the black fixation cross in the centre of the screen changed to either red or green, that is, press “D” when the cross was green and “K” when the cross was red. They were asked to respond as quickly and accurately as possible. There were two blocks. The key-color correspondence was swapped in the second block. Each trial started with a black fixation cross for 500 ms on a gray background. Then two copies of a character (font: Sinsum; size: 75 pixels × 75 pixels; color: black; duration: 150 ms) were presented symmetrically to the right and to the left of the cross (i.e., perifoveally). Following this were three 200 ms-long blank screens, in the middle of which the fixation cross color was randomly changed to green or red. Participants had to respond to the color changes. This was followed by another two-character perifoveal presentation. Finally, there was a blank screen with a random duration of 200–300 ms. Throughout the trial, fixation crosses were always presented in the middle of the screen in order to maintain participants’ attention to screen center and not to perifoveal stimuli.

#### EEG Data Recording

Brain Vision Recorder with a 32-channel EasyCap (Brain Products, Germany) (sampling rate 500 Hz, online band-pass filters at 0.01–100 Hz) was used to record electrophysiological (EEG) data. The reference electrode was attached to the tip of the nose. Horizontal and vertical electrooculography (EOG) were monitored using two electrodes placed on the outer canthi of the left eye and below the right eye, respectively. Impedances of all the electrodes (except EOGs which were below 10 kΩ), were maintained below 5 kΩ. In processing the data offline using Brain Vision Analyzer (Brain Products GmbH, Munich, Germany), a baseline of 100 ms prior to and 600 ms after the stimulus event was adopted. Butterworth Zero Phase digital shift (bandwidth 0.1–30 Hz, slope 24 dB/Octave), was used to filter data. Artifacts, including eye blinks, movement or muscle potentials, exceeding an absolute value of 100 μV at any electrode except EOGs were discarded. Four conditions of deviant concrete character, standard concrete character, deviant abstract character and standard abstract character were averaged, respectively, for further analysis. In the averaging procedure, the first three epochs in each block, and stimulus events preceded by a color fixation cross or by a button press were rejected. Data from four participants were discarded before proceeding to further statistical analysis, due to limited number of usable segments (<50% of all segments) in one of the four conditions, or low accuracy rate in the behavioral distraction task (<50%).

#### ERP Data Analysis

**FIGURE 2:**
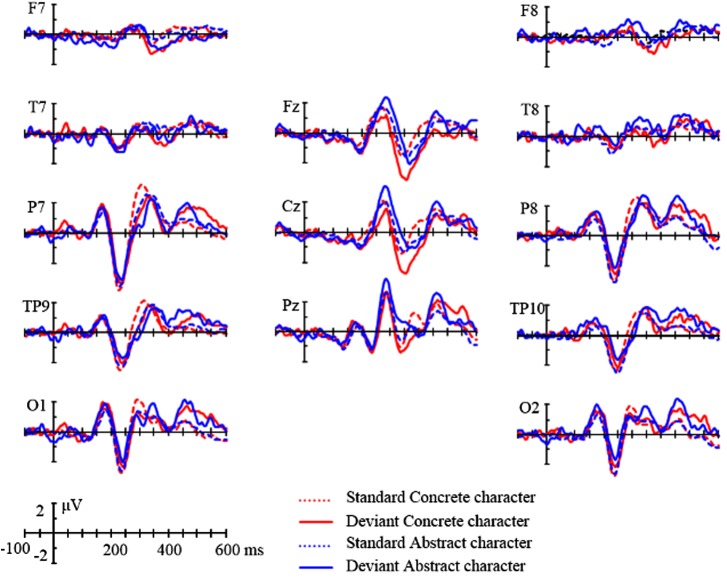
Comparison of standard and deviant stimuli. ERP waveforms for standard (red dotted) and deviant (red solid) concrete character conditions and for standard (blue dotted) and deviant (blue solid) abstract character conditions at midline electrodes Fz, Cz, and Pz and lateral electrodes F7/8, T7/8, P7/8, TP9/10, and O1/2. Negativity is plotted downward.

Choice of electrode sites for analysis was made with reference to previous literature on the semantic concreteness effect (West and Holcomb, 2000; Zhang et al., 2006) and on semantic MMN studies (Pulvermüller et al., 2001; Shtyrov et al., 2004). Midline and lateral site ERPs, quantified in respective areas by averaging the amplitudes of the electrodes included, were analyzed in ANOVAs of stimulus Type (deviant and standard), Condition (concrete and abstract) and Topographical factors. See Figure 2 for the ERPs of deviants and standards of the two word classes. At the midline sites, the topographical factor was Region, including frontal (Fz, FC1, and FC2), central (Cz, CP1, and CP2) and parietal (Pz, P3, and P4) areas. At the lateral sites, the topographical factors included Hemisphere (left and right) and Region: frontal (F7/8), temporal (T7/8), parietal (P7/8), temporo-parietal (TP9/10) and occipital (O1/2) sites. Time windows spanning a 40 ms interval from 200 to 440 ms were selected for analysis, except the two windows of 320–360 ms and 360–400 ms which were merged. These windows were selected with references to previous studies (West and Holcomb, 2000; Zhang et al., 2006). The dependent variable is mean amplitude of voltages averaged across the electrodes in the selected regions and time windows. In all of the statistical analyses, Greenhouse-Geisser correction of the degrees of freedom was applied and the corrected *p*-values are reported, where appropriate.

#### Time-Frequency Analysis

**FIGURE 3:**
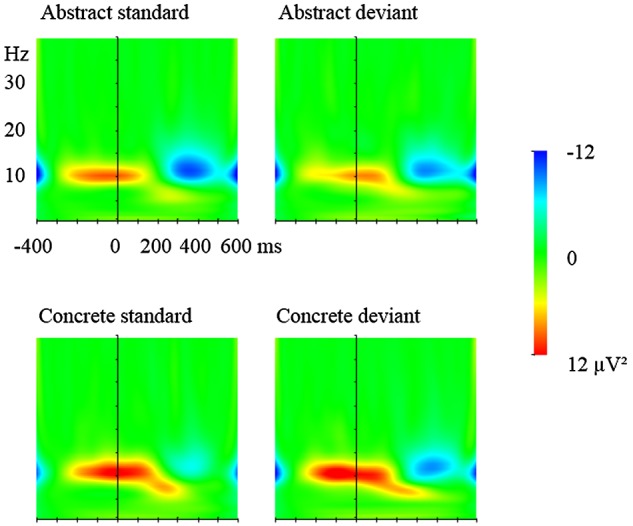
Event-related spectral perturbation (ERSPs) of standard and deviant words. **(Upper)** Standard and deviant abstract words; **(lower)**: standard and deviant concrete words. The plots are based on electrode P7.

**FIGURE 4:**
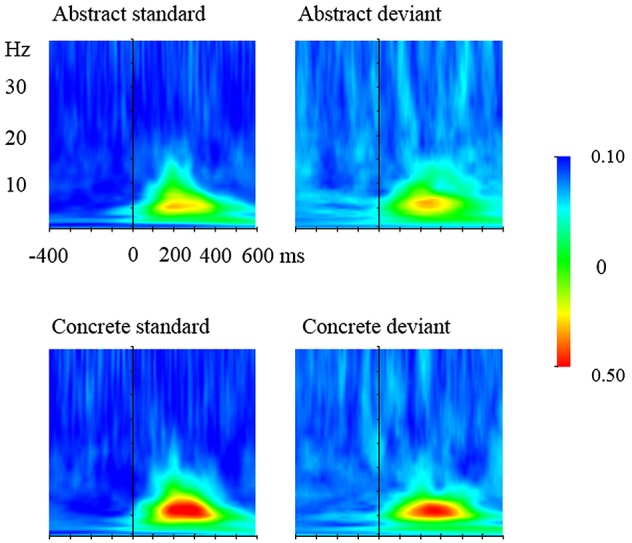
Phase-locking value (PLVs) of standard and deviant words. **(Upper)** Standard and deviant abstract words; **(lower)** standard and deviant concrete words. The plots are based on electrode P7.

To further uncover the oscillatory underpinnings of the mismatch responses, time-frequency analysis of the EEG data was conducted, also using Brain Vision Analyzer (Brain Products GmbH, Munich, Germany). Following the above-mentioned artifact rejection step in the EEG data processing, the four experimental conditions (Type: deviant and standard by Concreteness: concrete and abstract) were segmented with a baseline of 400 ms before the onset of stimuli. Here, the number of segments of standards was matched with that of deviants by selecting the standard stimuli preceding the deviant for analysis. This way, the potential influence of the number of the to-be-analyzed segments on TF representations, especially PLV, can be removed. Morlet wavelet transform was then applied to the segments with a frequency range from 1 to 40 Hz. In order to achieve a balance of time and frequency resolution (Tallon-Baudry, 2004), the Morlet parameter was set to 5. ERSPs that include both phase-locked and non-phase-locked oscillations were computed by averaging the absolute values of the wavelet transforms of segments, with a baseline correction based on the pre-stimulus interval from -400 ms to -200 ms (Figure 3). In calculating PLVs, the wavelet coefficients, instead of the spectral powers of the wavelet transform were measured first. PLVs were then computed and rectified before data extraction for statistical analysis (Figure 4).

## Results

### Behavioral Performance

Paired samples *t*-test yielded no difference between the two blocks in terms of reaction times in response to the central fixation color changes, in the block with concrete deviants (572 ms) and in the other block with abstract deviants (581 ms), *t*(20) = -1.32, *p* > 0.1. In terms of accuracy, paired samples *t*-test suggested slightly higher accuracy rate in the block with concrete standards (93.2%) than in the other block with abstract standards (90.2%), *t*(20) = -2.09, *p* < 0.05.

### ERP Results

As can be seen in Figure 2, both concrete and abstract words elicited canonical N/P1, N2 and P2 at most of the lateral sites. In addition, there is a noticeable divergence between concrete and abstract words when comparing their deviants and standards ERPs after 300 ms at midline sites. While for the concrete word deviants appeared to be more negative than standards over a time interval from 300 ms to around 450 ms, there is no such a sign for abstract words.

#### 200–240 ms

At lateral sites, ANOVA yielded an interaction between Type, Hemisphere and Region, (4,80) = 2.78, *p* = 0.05, $\eta_{p}^{2}$ = 0.12. *Post hoc* analysis indicated that deviants were more negative than standards only at left frontal site, -0.38 vs. 0.05 μV, *p* < 0.01. At midline sites, ANOVA yielded an interaction between Type and Region, *F*(2,40) = 6.46, *p* < 0.05, $\eta_{p}^{2}$ = 0.24. Further analysis did not show difference between deviants and standards in any of the three regions, *p*s > 0.1.

#### 240–280 ms

ANOVA at lateral sites indicated an interaction between Type and Hemisphere, *F*(1,20) = 5.66, *p* < 0.05, $\eta_{p}^{2}$ = 0.22. *Post hoc* analysis, however, did not indicate a difference between deviants and standards, *p*s > 0.1. At midline sites, there were no reliable effects.

#### 280–320 ms

At lateral sites, there was an interaction of Type and Region, *F*(4,80) = 10.36, *p* < 0.001, $\eta_{p}^{2}$ = 0.34. *Post hoc* analysis indicated that deviants were more negative than standards at the temporo-parietal (0.27 vs. 1.04 μV, *p* < 0.01) and parietal (1.14 vs. 2.15 μV, *p* < 0.01) regions. There was an interaction of Type and Hemisphere, *F*(20,1) = 5.55, *p* < 0.05, $\eta_{p}^{2}$ = 0.22. *Post hoc* analysis indicated more negativity to deviants than to standards in the left hemisphere, 0.59 vs. 1.26, *p* < 0.01. At midline sites, there were no reliable effects.

#### 320–400 ms

At lateral sites, ANOVA indicated an interaction of Type, Condition and Hemisphere, *F*(1,20) = 9.09, *p* < 0.01, $\eta_{p}^{2}$ = 0.31. *Post hoc* analysis, however, did not show a difference between deviant and standard stimuli. At midline sites, there was an interaction of Type and Region, *F*(2,40) = 5.12, *p* < 0.05, $\eta_{p}^{2}$ = 0.20. Further analysis showed that deviants were more negative than standards in the frontal region, -1.60 vs. -0.82, *p* < 0.05. There was also an interaction of Type and Condition, *F*(1,20) = 4.41, *p* < 0.05, $\eta_{p}^{2}$ = 0.18. Further analysis indicated that deviants were more negative than standards only for concrete words, -1.33 vs. -0.01, *p* < 0.05.

#### 400–440 ms

At lateral sites, ANOVA yielded an interaction of Type and Region, *F*(4,80) = 10.35, *p* < 0.001, $\eta_{p}^{2}$ = 0.34. *Post hoc* analysis indicated more negativity for deviants and standards in Frontal and Temporal regions, -0.22 vs. 0.26; 0.05 vs. 0.42, *p*s < 0.05. At midline sites, the interaction of Type, Condition and Region reached marginal significance, *F*(2,40) = 3.77, *p* = 0.056, $\eta_{p}^{2}$ = 0.16. Further analysis indicated that while deviants did not differ from standards for abstract words, *p* > 0.1, deviants were more negative than standards for concrete words in the frontal region, -0.51 vs. 0.84, *p* < 0.05.

### Time-Frequency Results

#### Event-Related Spectral Perturbation (ERSP)

##### 200–300 ms

Deviant and standard stimuli elicited an increase in overall spectral power, greatest at 6 Hz. In the lateral analysis, ANOVA yielded an interaction between Condition and Region, *F*(4,80) = 10.41, *p* = 0.001, $\eta_{p}^{2}$ = 0.33. *Post hoc* analysis revealed that concrete words produced more power increase than abstract words at the parietal and occipital sites, 5.16 vs. 3.83 μV^2^, 3.03 vs. 1.40 μV^2^, *p*s < 0.05. In the midline analysis, ANOVA yielded a main effect of Type, with deviant stimuli producing larger power increase than standard stimuli, *F*(1,20) = 9.81, *p* < 0.01, $\eta_{p}^{2}$ = 0.33, 3.64 vs. 1.90 μV^2^. There was also an interaction of Condition and Region, *F*(2,40) = 7.42, *p* < 0.01, $\eta_{p}^{2}$ = 0.27. *Post hoc* analysis, however, did not show any differences between the two word classes.

##### 300–450 ms

Both deviant and standard stimuli elicited a decrease in overall spectral power, greatest at 12 Hz (Figure 3). In the lateral analysis, ANOVA revealed an interaction between Type and Condition, *F*(1,20) = 4.80, *p* < 0.05, $\eta_{p}^{2}$ = 0.19. *Post hoc* analysis indicated that the difference between deviants and standards in concrete words reached marginal significance, *p* = 0.062, -2.58 vs. -0.95 μV^2^, while there was no difference between deviant and standard abstract words, -2.37 vs. -3.28, *p* > 0.1. The interaction of Condition and Region reached significance, *F*(4,80) = 4.16, *p* < 0.01, $\eta_{p}^{2}$ = 0.17. *Post hoc* analysis showed that abstract words had stronger power decrease than concrete words in temporal-parietal (-2.01 vs. -0.97 μV^2^, *p* < 0.05), parietal (-5.57 vs. -3.75 μV^2^, *p* < 0.05), and occipital (-6.36 vs. -4.18 μV^2^, *p* = 0.058) sites. In the midline analysis, no significant effects between standards and deviants were observed.

#### Phase-Locking Value (PLV)

Both deviant and standard stimuli elicited an increase in PLV, strongest at 4–8 Hz, between 150 and 450 ms (Figure 4). The increased PLV was focused in temporal and parietal areas. ANOVA was carried out separately for lateral and midline sites in the window of 200–450 ms. In the lateral analysis, ANOVA revealed an interaction between Type and Hemisphere, *F*(1,20) = 5.92, *p* < 0.05, $\eta_{p}^{2}$ = 0.23. *Post hoc* analysis found that deviants elicited higher PLV in the left hemisphere than in the right hemisphere, 0.29 vs. 0.26 μV. The interaction between Type and Region also reached significance, *F*(4,80) = 5.87, *p* = 0.01, $\eta_{p}^{2}$ = 0.23. *Post hoc* analysis showed that deviants elicited higher PLV in frontal and temporal sites, 0.24 vs. 0.19 μV, 0.22 vs. 0.18 μV, *p*s < 0.001. ANOVA also revealed an interaction between Condition and Region, *F*(4,80) = 5.22, *p* < 0.01, $\eta_{p}^{2}$ = 0.21. *Post hoc* analysis showed that concrete words had higher PLV than abstract words at temporal, temporal-parietal, parietal and occipital sites, 0.21 vs. 0.19 μV, *p* = 0.054, 0.33 vs. 0.30 μV, *p* < 0.01, 0.36 vs. 0.32 μV, *p* < 0.01, 0.29 vs. 0.27 μV, *p* < 0.05. In the midline analysis, ANOVA revealed a main effect of Type, *F*(1,20) = 12.27, *p* < 0.01, $\eta_{p}^{2}$ = 0.38. *Post hoc* analysis showed that deviants elicited larger PLV than standards, 0.27 vs. 0.24 μV. No other effects reached significance.

## Discussion

The current experiment investigated early and automatic processing of concreteness in Chinese character reading. Concrete and abstract characters were presented perifoveally and participants were asked to carry out a non-linguistic distraction task presented in the middle of the screen. An oddball paradigm was used, where concrete and abstract characters acted as deviants and standards in one block and were swapped in the other block. Deviants elicited larger negative amplitudes than standards in various time windows across 200–440 ms. In early intervals before 320 ms, both concrete and abstract words elicited more negativity to deviants than to standards (i.e., vMMNs) only at lateral sites, specifically, at left frontal (200–240 ms), temporal-parietal and parietal sites (280–320 ms). At 320–400 ms, only concrete words yielded vMMN in the midline regions. At 400–440 ms, both categories of words elicited vMMNs at lateral frontal and temporal sites, but only concrete words tended to show vMMN at the central-frontal area.

As for the TF results, in the interval of 200–300 ms, deviants elicited larger theta power increase, peaking around 6 Hz in the central areas. Concrete characters elicited a larger theta power increase than abstract words at the lateral sites. Then from 300 to 450 ms, deviant stimuli elicited larger alpha power decrease than standard stimuli at the lateral sites. Abstract characters elicited larger alpha power decrease than concrete characters in lateral sites (specifically, parietal, temporo-parietal and occipital sites). In terms of PLV, in the interval of 200–450 ms, deviants elicited larger PLV than standards at the lateral (frontal and temporal sites) and central area; and concrete characters yielded larger PLV than concrete characters mainly at temporal, parietal and occipital sties. These results are discussed below.

### Mismatch Responses of Concrete and Abstract Words

Before 320 ms, concrete and abstract word vMMNs had similar lateral distributions. According to the “spatial distinctiveness principle” (Holcomb et al., 1999), it can therefore be reasonably assumed that the two word categories have similar processing mechanisms. In these early windows, the negativities for both word classes are found to be left-lateralized, which may suggest that initially, verbal information is rapidly processed. Indeed, it has long been claimed that verbal systems are predominantly located in the left hemisphere (e.g., Coltheart et al., 1980; Chiarello et al., 1987; Kemmerer, 2014). Therefore, our data seem to be more in line with the dual-coding theory, which postulates a similar representational/verbal system for both concrete and abstract words.

After 320 ms, both concrete and abstract words yielded vMMNs at lateral frontal and temporal regions (400–440 ms). However, only concrete words additionally elicited vMMNs in the central area (320–400 ms) and middle frontal area (400–440 ms). This indicates that under non-attend conditions, concrete words still enjoy a semantic processing advantage, i.e., concreteness effect. This advantage in vMMN elicitation is arguably a result of the more perceptually salient referents of concrete words. These referents may contribute to a more robust and extensive neural network, and thus stronger memory representations than those of abstract words. Consequently, even in a context that discourages semantic processing, activation of concrete words can still be possible and is neurophysiologically reflected as the vMMN effect.

The concrete word vMMN with a frontal and central distribution is reminiscent of the well-documented concreteness N400 effects (Holcomb et al., 1999; West and Holcomb, 2000; Zhang et al., 2006; Kanske and Kotz, 2007; Tsai et al., 2009). The concreteness N400 effect is associated with more pronounced negativity over frontal sites, in contrast to the traditional semantic N400 effect characterized by centro-parietal morphology. Given the current non-attend oddball design, which does not encourage semantic processing, the emergent concreteness vMMNs suggest the resilient nature of the concreteness effect. In line with this, previous studies have also found concreteness N400 effects in tasks requiring various depths of semantic processing, such as image-generation (West and Holcomb, 2000), lexical decision (Tsai et al., 2009) or incidental memory retrieval (Nittono et al., 2002; Xiao et al., 2012). While the cortical generators of concrete words are strongly dependent on specific semantic categories (such as leg/arm/face-related words) (Hauk and Pulvermüller, 2004), some ERP studies on early semantic processing do find in source analyses that fronto-central areas are sensitive to form-related (Moscoso Del Prado Martín et al., 2006) and object-related (Moseley et al., 2013) words, which, to some extent, resemble the concrete words in the current study. Taken together, our data are consistent with previous studies and provide new evidence for distinct processing mechanisms responsible for abstract and concrete words. It should be noted, however, that possible parallels between the topographical distribution of ERP results and previous findings on semantic generators should be taken with caution due to the EEG inverse problem (Luck, 2005).

As discussed above, the similarities and differences from 200 to 440 ms between concrete and abstract words provide evidence for the dual-coding theory (Paivio, 1991). Our results therefore run against the context availability theory (Schwanenflugel and Shoben, 1983), according to which there should have been similar effects and scalp distributions for mismatch responses to both abstract and concrete words.

### Early and Automatic Semantic Change Detection Revealed by vMMNs

In this experiment, words were contrasted in terms of their concreteness, but matched in visual complexity (stroke numbers), phonetic regularity, lexical frequency and emotionality (valence and arousal). Therefore, the ERP difference between deviant and standard stimuli is best attributed to the semantic contrast in terms of concreteness ratings. In other words, the vMMNs were elicited by rapid and automatic detection of semantic change. This finding extends previous vMMN investigations of non-linguistic object feature detection (for a recent review, see Kremláček et al., 2016) to the higher-order semantic level. In terms of the earliness of vMMN effects, both abstract and concrete words elicited vMMNs as early as 200–240 ms, which is also in line with previous studies on semantic processing in the auditory MMN field (Shtyrov et al., 2004; Pulvermüller et al., 2005; Shtyrov, 2010) as well as studies using other paradigms such as RP (Hinojosa et al., 2004) and lexical decision (Sereno and Rayner, 2003).

In addition, these early semantic vMMN effects occurred despite the non-attend design, and with a distracting non-linguistic color-tracking task, suggesting that the semantic processing was automatic in nature. Recently, Fujimura and Okanoya (2013), in one of the few studies on semantic processing using a vMMN design, explored automatic detection of the emotional connotations of Kanji words using an oddball paradigm similar to that of the current study. They also described early, enhanced vMMN to a strongly emotional deviant in comparison with neutral standards, at an early latency of 200–300 ms. In their study, however, the targets in the distraction task also served as members of the vMMN-eliciting emotion words. In addition, all the stimuli were centrally presented, despite the fact that emotional connotation is particularly attention-capturing, due to its relevance to survival (Lang et al., 1997). It could be argued, therefore, that their experimental setting may not have been stringent enough to avoid the attention-grabbing effects of the vMMN-eliciting critical stimuli. Thus, the automatic nature of those findings may be questionable. In the current study, with an improved design of perifoveal presentation of critical stimuli, vMMNs were, however, also observed, providing more compelling evidence for automatic semantic change detection.

The earliness and automaticity of semantic change detection in the current study may be explained by the long-term memory trace theory of linguistic MMN effects (Pulvermüller and Shtyrov, 2006; Shtyrov, 2010). As all the participants in the current experiment are native Chinese college students, they presumably have developed strong mental representations of the word stimuli tested here, or in other words, long-term memory representations of the linguistic items, as suggested and confirmed in previous MMN studies with spoken word stimuli (Pulvermüller et al., 2009). Additionally, the unique features of Chinese words may also play a role in the earliness of the concreteness effect. Chinese words are characterized by direct orthography-meaning correspondence with a relatively weak mediating role of phonology in accessing word meaning (Chen and Shu, 2001; Wang, 2011; Zhang et al., 2012, but see Perfetti and Tan, 1998). Indeed, there is no direct grapheme-phoneme correspondence in the writing system of Chinese, in contrast to alphabetic languages such as English where letters have more or less clear phonemic representations. Of particular relevance here, Zhang et al. (2006) investigated concreteness effects in Chinese words in a lexical decision task. Apart from the typical N400 concreteness effect at 300–500 ms, at an earlier interval of 200–300 ms, a concreteness effect was also observed. Therefore, the characteristics of Chinese writing in terms of semantic activation may play a part in these early semantic vMMN effects.

### Oscillatory Characteristics

In the interval of 200–300 ms, both deviant and standard stimuli elicited overall spectral power increase in the theta range. This finding is in line with previous MMN studies in both auditory and visual modalities using elementary non-linguistic stimuli (Hsiao et al., 2009; Hsiao et al., 2010; Ko et al., 2012; Stothart and Kazanina, 2013), which have indicated the role of theta band oscillations in deviant detection, regardless of the mode of presentation. For example, in a vMMN study on frame color change detection, Stothart and Kazanina (2013) reported that similar theta power increase was yielded by both deviant and standard stimuli before 200 ms. In the current experiment, while at lateral sites the theta power between deviants and standards did not differ, deviants elicited larger theta power increase than standards in the central area. This difference between the two types of stimuli has seldom been reported in the above-mentioned MMN studies (Hsiao et al., 2009, 2010; Ko et al., 2012; Stothart and Kazanina, 2013). The reason for this may lie in the special stimulus category of language, compared to the basic-level auditory or visual features often targeted in those studies. Since deviant and standard words are different only in the dimension of semantic concreteness, the theta power difference may reflect such early semantic change detection. Consistent with this viewpoint, previous studies have indicated that theta band power increase can reflect lexical-semantic information retrieval (Bastiaansen et al., 2005, 2008). In fact, at parietal and occipital sites, the current experiment also found enhanced theta power in response to concrete words in comparison with abstract words, adding further evidence for the relevance of theta power to semantic processing.

In the following window of 300–450 ms, all the stimuli elicited alpha power decrease. Alpha power decrease has been reported in previous vMMN studies focusing on elementary visual feature detection (Stothart and Kazanina, 2013; Tugin et al., 2016). It has been suggested that while alpha power increase reflects task-related inhibition within a cortical area (Palva and Palva, 2007; Jensen et al., 2014), a decrease in the oscillatory amplitude suggests active neuronal processing (Pfurtscheller and Da Silva, 1999; Thut et al., 2012) and the enhanced attentional demands of processing (Klimesch, 1999; Bastiaansen et al., 2002; Stothart and Kazanina, 2013; Wang and Bastiaansen, 2014). Therefore, the alpha power decrease here might indicate that both deviants and standards are undergoing further processing after the initial early-phase semantic processing. Stothart and Kazanina (2013) and Tugin et al. (2016) reported a larger alpha reduction for deviants than standards, whereas in the current study there is no such effect. One possible reason for this discrepancy might again lie in the types of stimuli used in the different studies. Though a non-attend paradigm is common to all three studies, linguistic items are relatively more difficult to process as deviants and standards, and thus demand more attentional resources than basic visual features such as color bars (Stothart and Kazanina, 2013) and moving dots (Tugin et al., 2016). Interestingly, in the current study, when deviant and standard abstract words are compared with deviant and standard concrete words, the former elicited a larger reduction in alpha band than the latter. This aligns well with previous studies showing that alpha power decrease, especially in its upper band (i.e., above 11 Hz) suggests semantic information retrieval, which demands enhanced attentional resources (Klimesch et al., 1997; Pérez et al., 2012). In line with this viewpoint, abstract words are considered to have fewer underlying semantic nodes in comparison with concrete words (Xiao et al., 2012), therefore making them less likely to support involuntary semantic retrieval in attention-deprived experimental conditions. An alternative explanation for the larger alpha power decrease for abstract words consists in the greater attentional demands for abstract word processing without involving semantic retrieval (Bastiaansen and Hagoort, 2006). However, due to the closely related association between attentional processing and semantic retrieval (Li and Ren, 2012), it is difficult to dissociate these two possibilities and the difference in alpha power decrease between abstract and concrete words may reflect a combination of both attentional demands and semantic processing. Further studies are needed to investigate the nature of alpha power decrease in response to factors of attention and semantics.

### Relationship Between ERPs and TF Representations

While the TF data adds a new perspective to exploration of semantic change detection mechanisms, the temporal resolution of spectral power analysis is not as precise as that of ERP measurement, which means the onset and offset of the overall spectral power effects should be better taken as a rough estimation of relevant underlying neurophysiological activity (Clochon et al., 1996). Nevertheless, the spectral power latencies of theta and alpha bands largely overlap with the ERP effects in the current experiment. The initial theta power increase may correspond with the early automatic change detection as indexed by the early stage vMMN effects before 250 ms. The difference in the alpha power decrease effects between concrete and abstract words appears to overlap in timing with the difference in vMMN elicitation after 300 ms. However, it is the abstract words that yielded larger alpha power decrease compared to the concrete words. This points toward the notion that time-frequency analysis of the event-related EEG responses may characterize different neurophysiological mechanisms from traditional ERP analysis (Wang and Bastiaansen, 2014).

### Phase Synchronization

Different from spectral power analysis, phase locking analysis characterizes the synchronization between spatially disparate areas into transitory neural networks, thus providing a unique tool to probe into neuronal dynamics. In the current study, the theta phase locking for deviants was larger than that for standards between 200 and 450 ms. This result coincides to some extent with the differences between the deviant and standard stimuli in the visual evoked potentials (i.e., vMMN effects). Therefore, theta phase locking is suggested to play a role in the vMMN effects. The result agrees well with a previous vMMN study on visual color bar detection (Stothart and Kazanina, 2013) as well as a series of auditory MMN studies (Fuentemilla et al., 2008; Hsiao et al., 2009; Bishop and Hardiman, 2010; Ko et al., 2012), indicating that the common role of theta phase locking in generating (v)MMN is independent of stimulus type and presentation modality. While these studies feature larger theta phase locking for deviant than standard stimuli in the right hemisphere, a clear left-lateralization was found in the current experiment. This topographic difference may be attributed to the processing of linguistic stimuli, in contrast to the basic feature perception explored in those studies. Similarly, larger phase locking for deviants than standards was found at frontal, temporal and midline sites, which appears to be consistent with the topographies of the vMMN effects, adding further support for the role of phase locking in language-related vMMNs in the time domain. In addition, in the window of 200–450 ms, concrete words elicited higher theta phase locking than abstract words in the midline analysis, which seems to be in line with the concrete word advantage in vMMN elicitation after 320 ms. This pattern may be attributable to the denser semantic links underlying concrete words, which neurophysiologically are represented as stronger functional connections. Therefore, the concreteness effect in the time domain is, to some extent, supported from the perspective of theta phase locking in the frequency domain.

## Conclusion

The current study shows that both abstract and concrete words can be processed early and automatically as indexed by their elicited vMMNs. After 320 ms, a concreteness effect was observed, with only concrete words eliciting vMMNs in the middle areas, suggesting distinct processing routes for the two word types. Corroborating the concreteness effect in the time domain, concrete words elicited larger theta power increase and higher phase locking than did abstract words. Interestingly, abstract words yielded larger alpha power decrease in a later window after 300 ms, possibly resulting from the greater difficulty in processing abstract words in an attention-limited condition. Our study also shows the applicability of vMMN to semantic processing, especially at its early latency before 300 ms.

## Author Contributions

DW and MG-D conceived and designed the experiments. DW carried out experiments and analyzed data. MG-D contributed materials and recording tools. DW drafted the manuscript. MG-D gave critical revisions. DW and MG-D reviewed and approved the final manuscript.

## Conflict of Interest Statement

The authors declare that the research was conducted in the absence of any commercial or financial relationships that could be construed as a potential conflict of interest.
